# circRNA DENND1B inhibits tumorigenicity of clear cell renal cell carcinoma via miR-122-5p/TIMP2 axis

**DOI:** 10.1515/med-2022-0536

**Published:** 2022-12-16

**Authors:** Deqiang Chen, Yanchun Zhang, Liang Meng, Li Lu, Gaopei Meng

**Affiliations:** Department of CT Diagnosis, Cangzhou Central Hospital, Hebei 061001, China; Department of Rehabilitation, Cangzhou Central Hospital, No. 16 Xinhua West Road, Cangzhou, Hebei 061001, China

**Keywords:** circ_DENND1B, miR-122-5p, TIMP2, ccRCC

## Abstract

Clear cell renal cell carcinoma (ccRCC) is the most common type of renal cancers. However, circ_DENND1B has not been studied yet. GSE100186 dataset was used for the level analysis of circ_DENND1B. The quantitative real-time PCR was used to verify the expression of circ_DENND1B, microRNA-122-5p (miR-122-5p) and tissue inhibitor of metalloproteinases-2 (TIMP2) in ccRCC tissues and cells. Cell proliferation, migration, invasion and apoptosis were detected by colony formation assay, thymidine analog 5-ethynyl-2′-deoxyuridine assay, 3-(4,5-dimethylthiazol-2-y1)-2,5-diphenyl tetrazolium bromide, transwell and flow cytometry. The binding of miR-122-5p to circ_DENND1B/TIMP2 was investigated by dual-luciferase reporter assay. Finally, the role of circ_DENND1B in ccRCC was detected by tumorigenesis experiment in mice. circ_DENND1B was downregulated in ccRCC and circ_DENND1B overexpression suppressed the malignant behaviors of ccRCC cells. circ_DENND1B acted as a sponge of miR-122-5p. miR-122-5p upregulation reversed the effects of circ_DENND1B on cell proliferation, migration, invasion and apoptosis. TIMP2 was a target of miR-122-5p. Overexpression of circ_DENND1B regulated TIMP2 level by inhibiting miR-122-5p expression in ccRCC cells. circ_DENND1B overexpression inhibited the tumor growth of ccRCC *in vivo*. circ_DENND1B inhibited ccRCC cell progression by promoting TIMP2 expression by sponging miR-122-5p, suggesting that circ_DENND1B might be an effective therapeutic target for ccRCC.

## 1 Introduction

Clear cell renal cell carcinoma (ccRCC) is the most representative subtype of renal cancer, accounting for 70–80% of all renal cancer types [1]. Surgical resection is still the best treatment strategy for ccRCC. However, most patients are diagnosed at advanced ccRCC because there are few biomarkers for the early diagnosis of ccRCC [2]. Patients at advanced stage are prone to recur due to cancer metastasis after surgery, and the 5-year survival rate is only 10–20% [3,4]. Therefore, it is particularly important to investigate the pathogenesis of ccRCC and find effective molecular targets for early diagnosis.

Circular RNA (circRNA) is a special RNA type with circular structure that differs from linear RNA in that it lacks a 5′cap and 3′tail [5]. circRNA is a non-coding RNA, is not easily degraded by RNA exonucleases and has high stability [6]. Meanwhile, circRNAs have been reported to act as competitive endogenous RNA (ceRNAs) and regulate gene expression through sponging microRNA (miRNA) [7,8]. In recent years, it has been found that aberrant expression of circRNA can be involved in regulating changes in ccRCC tumor and cell biology by targeting miRNA/mRNA [9]. For instance, Liu et al. found that circ_0085576 regulated YAP1 signaling pathway in ccRCC by targeting miR-498 [10]. circ_RPL23A exerted oncogenic function through mediating miR-1233/ACAT2 axis [11]. Cheng et al. revealed that circSDHC promoted RCC metastasis by competitively binding to miR-127-3p and regulating CDKN/E2F1 axis [11].

In this study, we obtained from GEO database GSE100186 data analysis that circ_DENND1B was significantly differentially expressed in ccRCC. microRNA-122-5p (miR-122-5p) was predicted to be the target of circ_DENND1B through bioinformatics website, and it directly targets the tissue inhibitor of metalloproteinases-2 (TIMP2). miR-122-5p has been reported as a cancer-promoting molecular marker in various cancers, such as gastric cancer, cervical cancer, pancreatic cancer and renal cancer [12,13,14,15]. Previous studies have shown that TIMP2 levels increased in acute kidney injury, and downregulation of TIMP2 ameliorated renal injury by inhibiting the NF-κB pathway by exerting anti-inflammatory activity [16]. TIMP2 acted as a tumor suppressor in RCC, and TIMP2 is targeted by miR-221 to regulate 786-O cell proliferation [17].

Herein, we aimed to investigate whether circ_DENND1B inhibited ccRCC progression through miR-122-5p/TIMP2 axis.

## 2 Materials and methods

### 2.1 Clinical tissue samples

Thirty-three cases of ccRCC tissues and paracancerous tissues that underwent surgical resection in Cangzhou Central Hospital were selected. All patients did not receive other therapies such as chemotherapy before surgery. The specimens were placed in liquid nitrogen and stored in an ultra-low temperature refrigerator at −80°C immediately after isolation.


**Ethics approval and consent to participate:** The present study was approved by the ethical review committee of Cangzhou Central Hospital. Written informed consent was obtained from all enrolled patients.
**Consent for publication:** Patients agree to participate in this work.

### 2.2 Cell culture and transfection

The renal cancer cell lines (786-O and 769-P) and human renal epithelial cells HK-2 obtained from ATCC (Manassas, VA, USA) were cultured in dulbecco’s modified eagle medium(DMEM) containing 10% fetal bovine serum (FBS) at 37°C. When cell density reached 60%, circ_DENND1B overexpression vector and blank vector, miR-122-5p mimics and inhibitors (anti-miR-122-5p) and their negative control (miR-NC/anti-miR-NC), siRNA targeted knockdown TIMP2 (si-TIMP2) and si-NC obtained from Ribobio Co., Ltd. (Guangzhou, China) were transfected into renal carcinoma cells using Lipofectamine 2000 (Thermo Fisher).

### 2.3 Quantitative real-time PCR (qRT-PCR)

TRIzol method was used to extract total RNA from ccRCC tissues, paracancer tissues, 786-O and 769-P cells and mouse tumor tissues used in this study. After the concentration and purity of total RNA were determined by UV spectrophotometer, the total RNA was reversely transcribed into cDNA. SYBR Green qRT-PCR Mix (Takara, Shiga, Japan) and Real-Time PCR Detection System (Bio-Rad, Shanghai, China) were used to detect the expression levels of related genes. GAPDH was selected as the internal reference gene of circ_DENND1B and TIMP2, and U6 was used as the internal reference gene of miR-122-5p. The relative expression was calculated by 2^−ΔΔCT^. The primers were available in Table 1.

**Table 1:** Primer sequences for qRT-PCR

Name	Primers (5′−3′)	
circ_DENND1B (hsa_circ_0000168)	Forward	TGAGCCCATCACTTTCTGT
	Reverse	TTGGGAGTCCAGTTACATCA
DENND1B	Forward	GCTGACAATGTGAGTGACCCTAC
	Reverse	CTCTTTGGCTTCTTTCCCTTCTT
TIMP2	Forward	CTGAGCACCACCCAGAAGAAGAGC
	Reverse	TGTGACCCAGTCCATCCAGAGGC
miR-122-5p	Forward	GTATGATGGAGTGTGACAA
	Reverse	TGGTGTCGTGGAGTCGT
GAPDH	Forward	AAGGCTGTGGGCAAGGTCATC
	Reverse	GCGTCAAAGGTGGAGGAGTGG
U6	Forward	CTCGCTTCGGCAGCACATA
	Reverse	CGAATTTGCGTGTCATCCT
circ_003997 (hsa_circ_0003997)	Forward	GCAACGAAGCTGGGAAGGAA
	Reverse	GGAAATTGGAAGCAAAGGCC
circ_092523 (hsa_circ_0001225)	Forward	CGAGCAGAGCGAGGAGAA
	Reverse	CCAACCGCAGCTTGGCAT
circ_400027 (hsa_circ_0092367)	Forward	CCAGAGGCTGTTGGGATC
	Reverse	TGACGGTATAGAGTTTTC
circ_000781 (hsa_circ_0000223)	Forward	AGGGAAGCTCAGTACGT
	Reverse	CACCGCTCCGCTGCCAG
circ_001654 (hsa_circ_0001605)	Forward	TTTTGAAGCGATGAGGTGT
	Reverse	GAAGAGGCAATGAAGGAAG

### 2.4 Western blot and immunohistochemistry (IHC) analysis

Western blot and IHC were performed using traditional methods [18]. The used antibodies were as follows: anti-GAPDH (1:1,000, ab9485; Abcam, Cambridge, MA, USA), anti-Proliferating Cell Nuclear Antigen (PCNA; 1:1,000, ab18197; Abcam), anti-Epithelial cadherin (E-cadherin; 1:1,000, ab231303; Abcam), anti-Twist1 (1:1,000, ab50887; Abcam), anti-TIMP2 (1:1,000, ab230511; Abcam) and anti-Ki67 (1:200, ab15580; Abcam).

### 2.5 circRNA validation

To block transcription, 786-O and 769-P cells were treated with 2 mg/mL actinomycin D (Act D; Sigma-Aldrich, St. Louis, MO, USA). qRT-PCR was used to determine the expression levels of circ_DENND1B and DENND1B mRNA.

### 2.6 Cellular distribution analysis

RNA was extracted according to the manufacturer’s instructions for NE-PER Nuclear and Cytoplasmic Extraction Reagent (Thermo Fisher). qRT-PCR was used to detect circ_DENND1B, U6 and GAPDH levels in cytoplasm or nucleus.

### 2.7 3-(4,5-Dimethylthiazol-2-yl)-2,5-diphenyltetrazolium bromide (MTT) assay

The treated cells were inoculated into 96-well plates and cultured in DMEM (containing 10% FBS) for 24 h. After 24 h, MTT solution (Sigma-Aldrich) was added to the wells, shaken well and incubated for 4 h in a 5% CO_2_ incubator at 37°C. 100 μL DMSO was added to each well and the wells were shaken for 10 min until the formazan were completely dissolved. The absorbance of each well was measured at 570 nm with an enzyme analyzer, and the absorbance represented the cell proliferation level.

### 2.8 Colony formation assay

786-O and 769-P were inoculated in six-well plates and cultured for 2 weeks, then fixed with 4% paraformaldehyde and stained with 0.4% crystal violet. Colonies of more than 50 cells were counted under a microscope.

### 2.9 Thymidine analog 5-ethynyl-2′-deoxyuridine (EdU) assay

Proliferation of 786-O and 769-P cells after treatment was detected using the Cell-Light EdU DNA Cell Proliferation Kit (RiboBio) according to the manufacturer’s instructions.

### 2.10 Transwell assay

The transwell chambers (Corning, New York, Madison, USA) were first added with diluted matrigel in the upper chamber (this step was not required for the determination of cell migration ability), and then, cells were re-suspended with serum-free DMEM and inoculated in the upper chamber. DMEM medium containing 10% FBS (500 μL) was added to the lower chambers for 6 h, then cells were fixed with 4% paraformaldehyde was used to fix the migrated and invaded cells in the lower chamber, and five fields were randomly selected for counting under the microscope after crystalline violet staining.

### 2.11 Flow cytometry assay

The single-cell suspension of 786-O and 769-P cells was adjusted to 1 × 10^6^ cells/mL using binding buffer. 100 μL cell suspension was added into the flow tube, Annexin-FITC and Propidium iodide (PI, BD Biosciences, San Diego, CA, USA) staining solution was added, and cell apoptosis rate was detected by flow cytometry for 15 min.

### 2.12 Dual-luciferase reporter assay

The binding site of circ_DENND1B and miR-122-5p was predicted in Circbank (http://www.circbank.cn/) website. The predicted circ_DENND1B sequence fragment was cloned into the PMIR-report vector, and two reporter plasmids circ_DENND1B-WT and circ_DENND1B-MUT were constructed. The reporter plasmid and miR-122-5p mimic ormiR-NC were co-transfected into 786-O and 769-P cells, and the luciferase activity was detected using Dual-Luciferase Reporter Assay Kit (GeneCopoeia, Rockville, MD, USA). The starbase (http://starbase.sysu.edu.cn/starbase2/) website was used to predict the binding sequence of miR-122-5p and TIMP2 and constructed two reporter plasmids TIMP2-WT and TIMP2-MUT. The target relationship between miR-122-5p and TIMP2 was also confirmed as above.

### 2.13 Xenograft models

All animal experiments were performed in accordance with the protocols approved by Cangzhou Central Hospital Animal Care and Use Committee. Five-week-old male BALB/C nude mice were purchased from Beijing Weidahe Laboratory Animal Science and Technology Co., Ltd. (Beijing, China). In tumor-forming experiments, 786-O cells stably transfected with circ_DENND1B overexpression vector (lenti_circ_DENND1B) or control vector (lenti_NC) were subcutaneously injected into mice (*n* = 5). After injection for 10 days, the length (*L*) and width (*W*) of tumors were measured with caliper every 5 days. Tumor size was calculated using the formula: tumor volume (*V*) = *L* × *W* ^2^/2. Thirty days later, the mice were euthanized and the tumors were weighed. qRT-PCR, western blot and IHC were performed on the transplanted tumor tissues.

### 2.14 Statistical analysis

SPSS 20.0 statistical software analyzed the data. Student’s *t*-test or one-way analysis of variance was used for the comparison of differences between two groups or multiple groups. All experiments were repeated three times. Using *P* < 0.05 meant a significant difference.

## 3 Results

### 3.1 circ_DENND1B was downregulated in ccRCC

**Figure 1:**
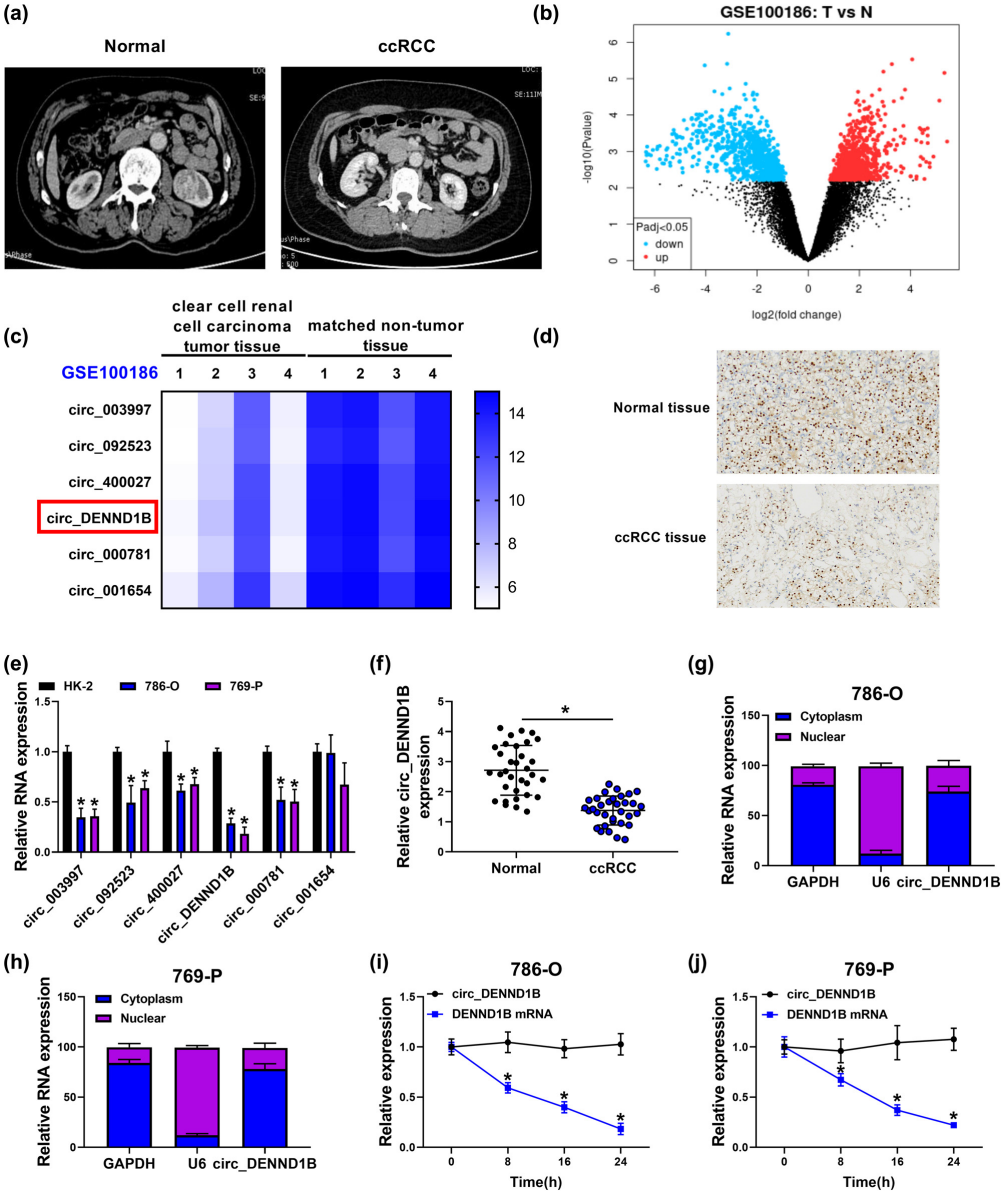
circ_DENND1B was downregulated in ccRCC. (a) Computed tomography scan of ccRCC tissues and normal tissues. (b and c) GSE100186 data were used to analyze the differential expression of circRNAs in renal cancer tissues. (d) IHC detected the positive cell rate of Ki67 in ccRCC tissues. (e) qRT-PCR detected the relative circRNA expression. (f) qRT-PCR detected the expression of circ_DENND1B in ccRCC cells. (g and h) The distribution of circ_DENND1B in nucleus or cytoplasm was determined by qRT-PCR. (i and j) Act D treated 786-O and 769-P cells and qRT-PCR detected circ_DENND1B and DENND1B mRNA levels. *P < 0.05.

Imaging showed that ccRCC tissue was significantly enlarged compared with normal renal tissue (Figure 1a). Analysis of GEO database GSE100186 data showed that the most significant 6 circRNAs were downregulated in ccRCC (Figure 1b and c). The rate of Ki67 positive cells was significantly reduced in ccRCC tissues detected by IHC (Figure 1d). The expression levels of 6 circRNAs in normal cells and ccRCC cells were detected by qRT-PCR, and we found that the differential multiple of circ_DENND1B expression was the highest (Figure 1e). Similarly, circ_DENND1B was downregulated in ccRCC cells (Figure 1f). In addition, the results of nucleo-cytoplasmic separation showed that circ_DENND1B was mainly distributed in the cytoplasm of 786-O and 769-P cells (Figure 1g and h). Furthermore, qRT-PCR analysis after Act D treatment confirmed that circ_DENND1B was a circRNA with stability (Figure 1i and j).

### 3.2 Overexpression of circ_DENND1B suppressed cell proliferation, migration and invasion of ccRCC cells

**Figure 2:**
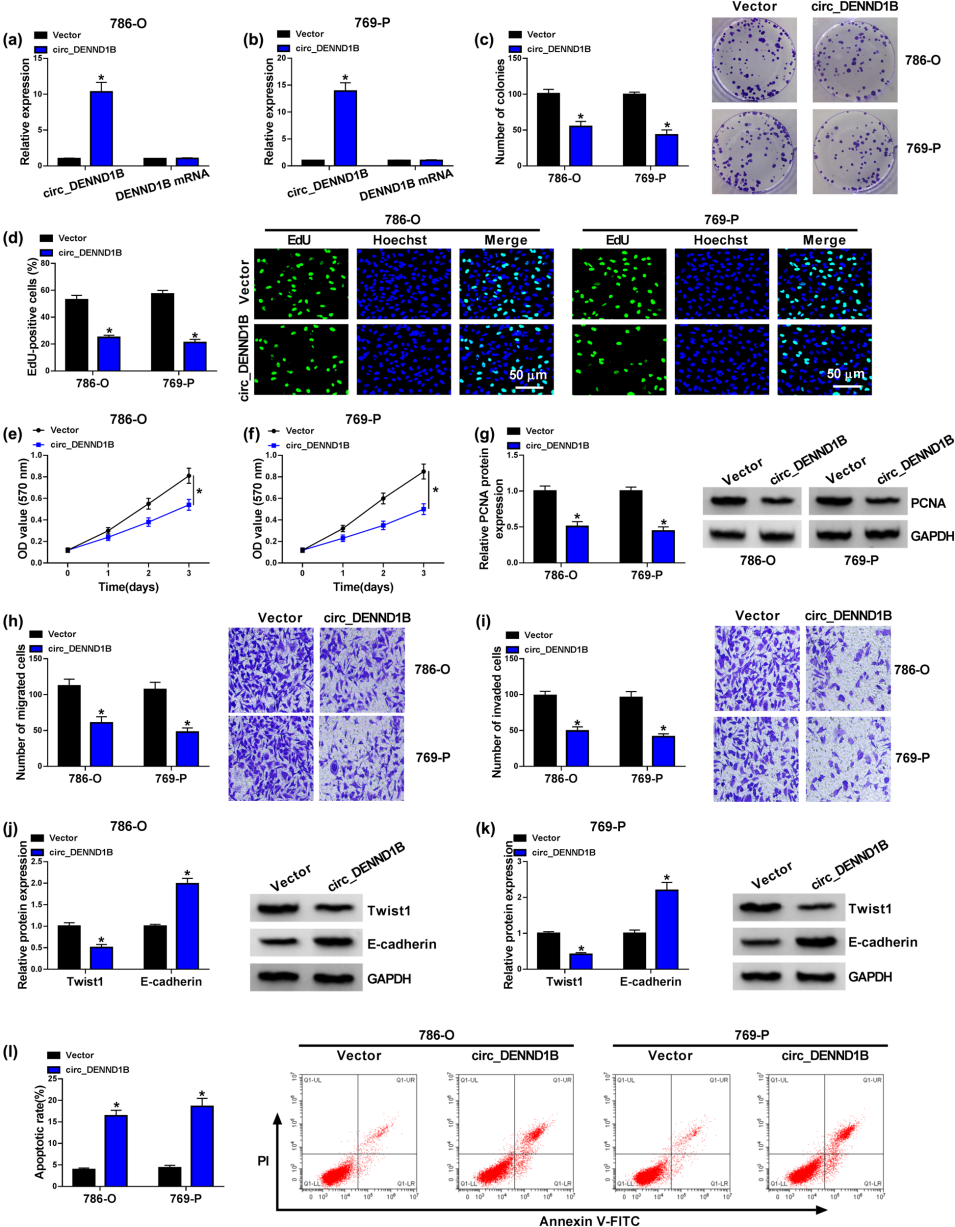
Overexpression of circ_DENND1B suppressed proliferation, migration and invasion of ccRCC cells. (a and b) qRT-PCR detected the expression of circ_DENND1B and DENND1B mRNA. (c) Colony formation assay detected the number of colonies. (d) EdU assay detected the EdU-positive cells. (e and f) MTT detected the cell viability. (g) Western blot detected the level of PCNA protein. (h and i) Transwell assay detected the cell migration and invasion ability. (j and k) Western blot detected the level of Twist1 and E-cadherin. (l) Cell apoptosis was detected by flow cytometry. *P < 0.05.

To investigate the role of circ_DENND1B in ccRCC, circ_DENND1B overexpression vector was transfected into 786-O and 769-P cells. The qRT-PCR assay showed that the level of circ_DENND1B was significantly upregulated in ccRCC cells after the transfection of circ_DENND1B overexpression vector, while there was no effect on the expression of DENND1B mRNA (Figure 2a and b). Cell proliferation was detected by colony formation assay and EdU assay. The results showed that overexpression of circ_DENND1B significantly reduced the proliferation of 786-O and 769-P cells (Figure 2c and d). Besides, the result of MTT assay showed that the cell viability of 786-O and 769-P cells was reduced after circ_DENND1B overexpression (Figure 2e and f). Similarly, upregulation of circ_DENND1B significantly inhibited the proliferation-related protein PCNA level (Figure 2g). Functionally, cell migration and invasion abilities of 786-O and 769-P cells were suppressed in the circ_DENND1B overexpression group compared with the vector group (Figure 2h and i). In addition, western blot detection of epithelial–mesenchymal transition (EMT)-related proteins showed that overexpression of circ_DENND1B decreased Twist1 expression and increased E-cadherin expression levels (Figure 2j and k). Conversely, circ_DENND1B upregulation notably increased cell apoptosis rate in 786-O and 769-P cells (Figure 2l). These data suggested that abnormal upregulation of circ_DENND1B could inhibit the progression of ccRCC cells.

### 3.3 circ_DENND1B acted as a sponge for miR-122-5p

**Figure 3:**
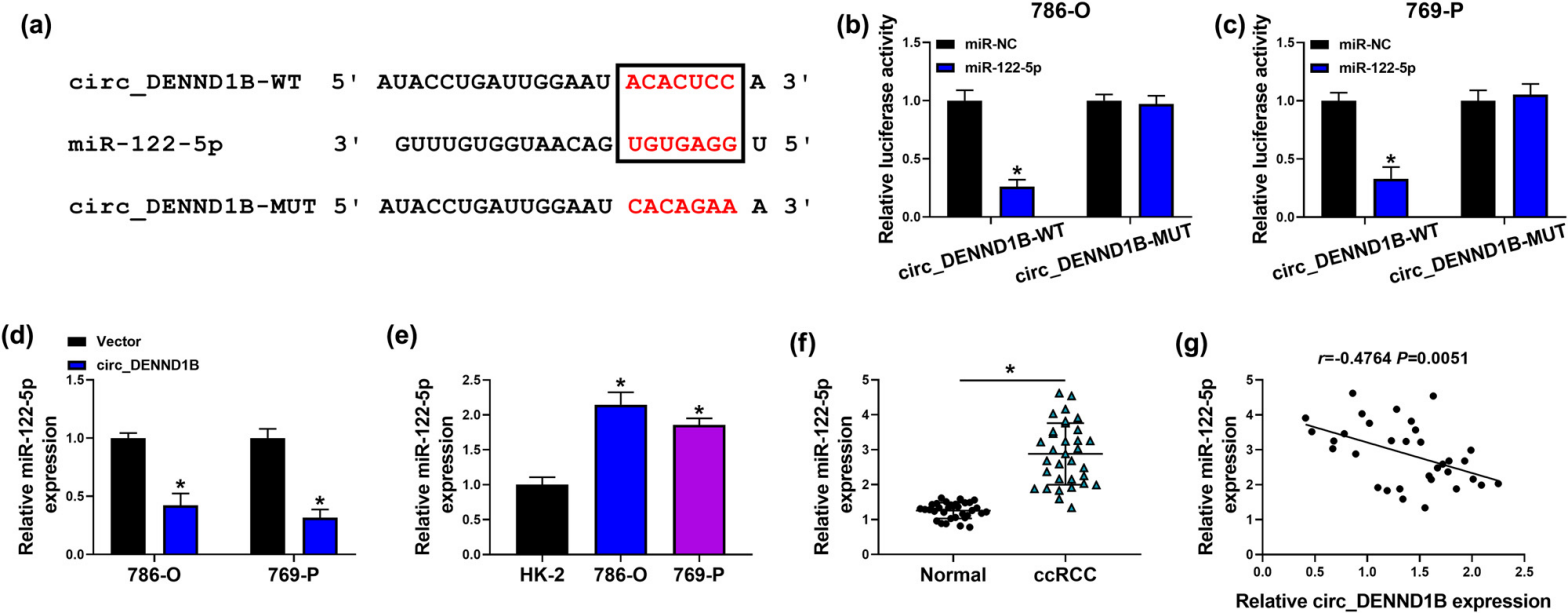
miR-122-5p was a target of circ_DENND1B. (a) The binding sites of circ_DENND1B to miR-122-5p. (b and c) Dual-luciferase reporter assays were performed to confirm the association between circ_DENND1B and miR-122-5p. (d–f) qRT-PCR detected the expression of miR-122-5p. (g) Pearson’s correlation analysis. *P < 0.05.

CircBank prediction indicated that miR-122-5p functioned as the target of circ_DENND1B with complementary binding sites (Figure 3a). Next, dual-luciferase assay was used to verify the binding of circ_DENND1B and miR-122-5p. The results showed that the luciferase activity was significantly reduced in ccRCC cells co-transfected with miR-122-5p mimics and circ_DENND1B-WT, but not with miR-122-5p mimics and circ_DENND1B-MUT (Figure 3b and c). Moreover, upregulated circDENND1B obviously decreased the expression of miR-122-5p (Figure 3d). Then, qRT-PCR demonstrated that miR-122-5p was greatly elevated in ccRCC cells and tissues (Figure 3e and f). Pearson’s correlation analysis confirmed that circDENND1B was negatively correlated with miR-122-5p (Figure 3g). These results suggested that miR-122-5p was a target of circ_DENND1B in ccRCC.

### 3.4 circ_DENND1B/miR-122-5p regulated the progress of ccRCC cells

**Figure 4:**
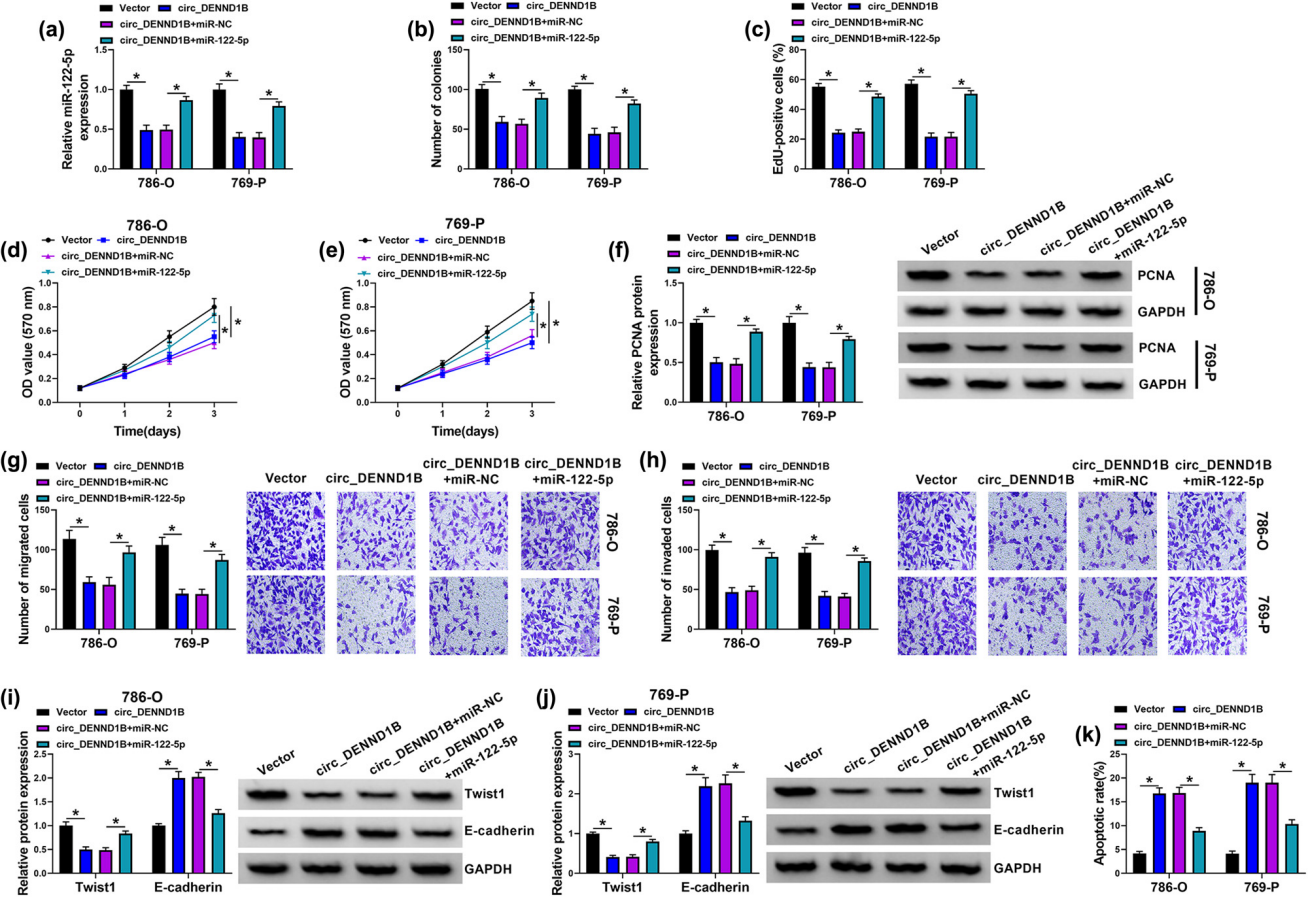
circ_DENND1B/miR-122-5p regulates progress of ccRCC cells. (a) qRT-PCR detected the expression of miR-122-5p. (b) Colony formation assay detected the number of colonies. (c) EdU assay detected the EdU-positive cells. (d and e) MTT detected the cell viability. (f) Western blot detected the level of PCNA protein. (g and h) Transwell assay detected the cell migration and invasion ability. (i and j) Western blot detected the level of Twist1 and E-cadherin. (k) Cell apoptosis was detected by flow cytometry. *P < 0.05.

The qRT-PCR showed that overexpression of circ_DENND1B inhibited miR-122-5p levels, while co-transfection of miR-122-5p mimics restored miR-122-5p levels (Figure 4a). Functionally, miR-122-5p reversed the cell proliferation inhibited by overexpressed circ_DENND1B (Figure 4b and c). Moreover, cell viability inhibition by circ_DENND1B was reversed after transfection with miR-122-5p mimics (Figure 4d and e). Also, miR-122-5p recovered the expression of PCNA protein repressed by circ_DENND1B (Figure 4f). In addition, the inhibitory effect of circ_DENND1B on cell migration and invasion was reversed after miR-122-5p co-transfection (Figure 4g and h). Overexpression of circ_DENND1B significantly decreased Twist1 expression and increased E-cadherin expression, while all of them were counteracted by miR-122-5p upregulation (Figure 4i and j). Mechanically, the promoting effect of circ_DENND1B on cell apoptosis was reversed in 786-O and 769-P cells co-transfected with miR-122-5p mimics (Figure 4k). In general, circ_DENND1B suppressed ccRCC cell progression via inhibiting miR-122-5p.

### 3.5 TIMP2 was a target of miR-122-5p

**Figure 5:**
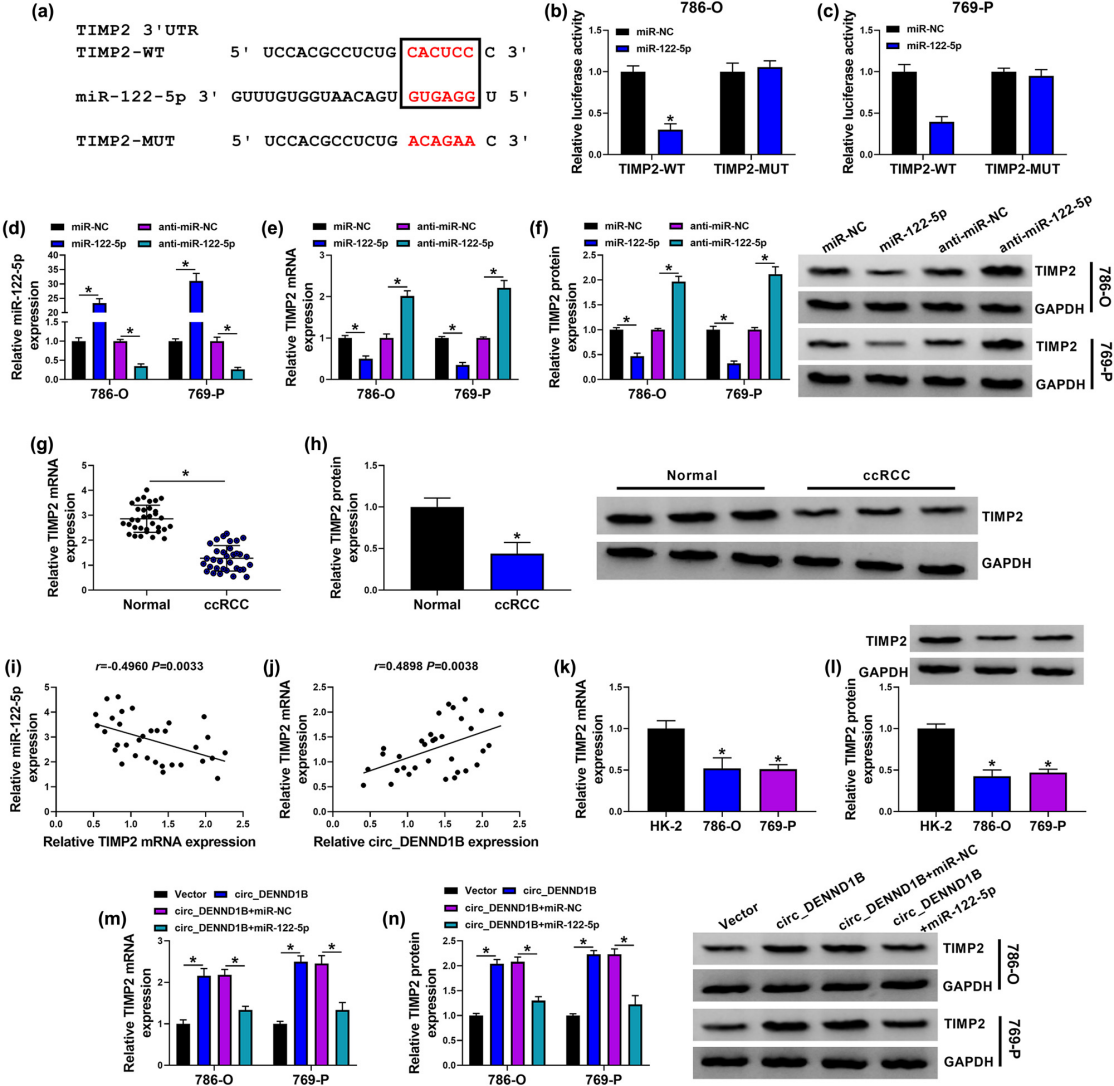
TIMP2 was a target of miR-122-5p. (a) The binding sites of miR-122-5p and TIMP2. (b and c) Dual-luciferase reporter assays were performed to confirm the association between TIMP2 and miR-122-5p. (d, e, g, k and m) qRT-PCR detected the expression of miR-122-5p and TIMP2. (f, h, l and n) Western blot detected the expression of TIMP2 protein. (i and j) Pearson’s correlation analysis. *P < 0.05.

Starbase predicted the targeted binding of miR-122-5p to TIMP2 (Figure 5a). Then, luciferase activity was significantly reduced in 786-O and 769-P cells co-transfected with miR-122-5p mimics and TIMP2-WT, while luciferase activity in 786-O and 769-P cells containing TIMP2-MUT was not affected by miR-122-5p (Figure 5b and c). Next, the overexpressed and inhibited efficiencies of miR-122-5p were detected by qRT-PCR in 786-O and 769-P cells, and the results showed that the expression of miR-122-5p was significantly upregulated after transfection with miR-122-5p mimic in ccRCC cells, while the level of miR-122-5p was significantly downregulated after transfection with anti-miR-122-5p (Figure 5d). Furthermore, miR-122-5p mimic significantly inhibited the expression of TIMP2 while anti-miR-122-5p promoted the expression of TIMP2 (Figure 5e and f). The qRT-PCR and western blot detected that TIMP2 was lowly expressed in ccRCC tissues (Figure 5g and h). Pearson’s correlation analysis showed that TIMP2 was negatively correlated with miR-122-5p and positively correlated with circ_DENND1B (Figure 5i and j). Similarly, TIMP2 was downregulated in ccRCC cells compared with HK-2 cells (Figure 5k and l). Functionally, miR-122-5p mimics downregulated the TIMP2 level elevated by circ_DENND1B (Figure 5m and n).

### 3.6 miR-122-5p/TIMP2 regulated the progress of ccRCC cells

**Figure 6:**
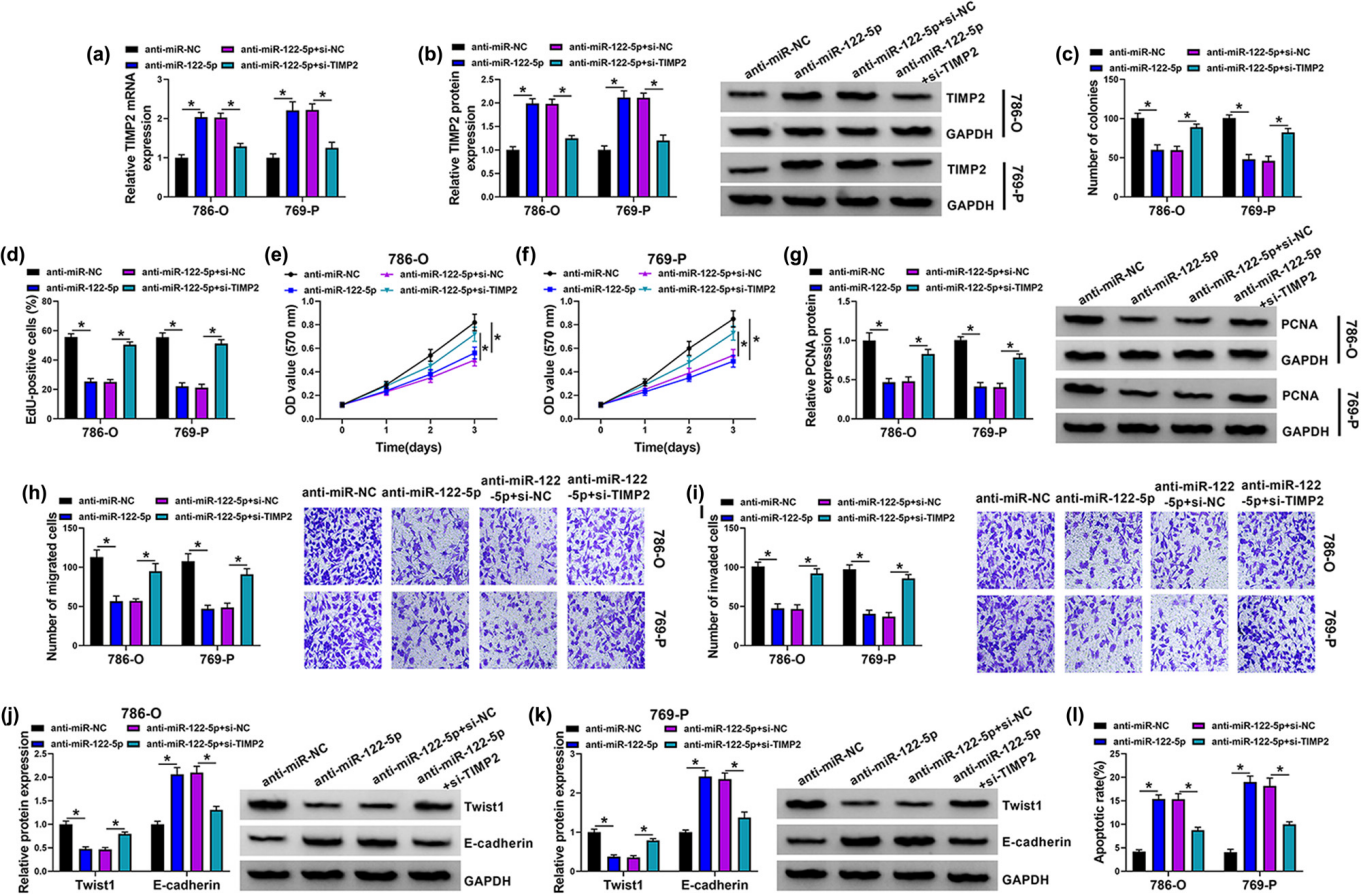
miR-122-5p/TIMP2 regulates progress of ccRCC cells. (a) qRT-PCR detected the expression of TIMP2. (b) Western blot detected the expression of TIMP2 protein. (c) Colony formation assay detected the number of colonies. (d) EdU assay detected the EdU-positive cells. (e and f) MTT detected the cell viability. (g) Western blot detected the level of PCNA protein. (h and i) Transwell assay detected the cell migration and invasion ability. (j and k) Western blot detected the level of Twist1 and E-cadherin. (l) Cell apoptosis was detected by flow cytometry. *P < 0.05.

First, qRT-PCR and western blot detected that anti-miR-122-5p promoted TIMP2 levels, while co-transfection of si-TIMP2 restored it (Figure 6a and b). Functionally, si-TIMP2 reversed cell proliferation inhibition by anti-miR-122-5p (Figure 6c and d). Moreover, the inhibitory effect of anti-miR-122-5p on cell viability was offset by si-TIMP2 (Figure 6e and f). Transfection of si-TIMP2 recovered the expression of PCNA protein suppressed by anti-miR-122-5p (Figure 6g). In addition, the inhibitory effect of anti-miR-122-5p on cell migration and invasion was reversed after si-TIMP2 co-transfection (Figure 6h and i). Similarly, anti-miR-122-5p notably decreased Twist1 expression and increased E-cadherin level, while all of them were reversed by si-TIMP2 (Figure 6j and k). Mechanically, the promoting effect of anti-miR-122-5p on cell apoptosis was reversed by co-transfected with si-TIMP2 in 786-O and 769-P cells (Figure 6l). In general, silencing TIMP2 reversed the effect of anti-miR-122-5p on 786-O and 769-P cells.

### 3.7 Overexpressed circ_DENND1B inhibited tumor growth *in vivo*

**Figure 7:**
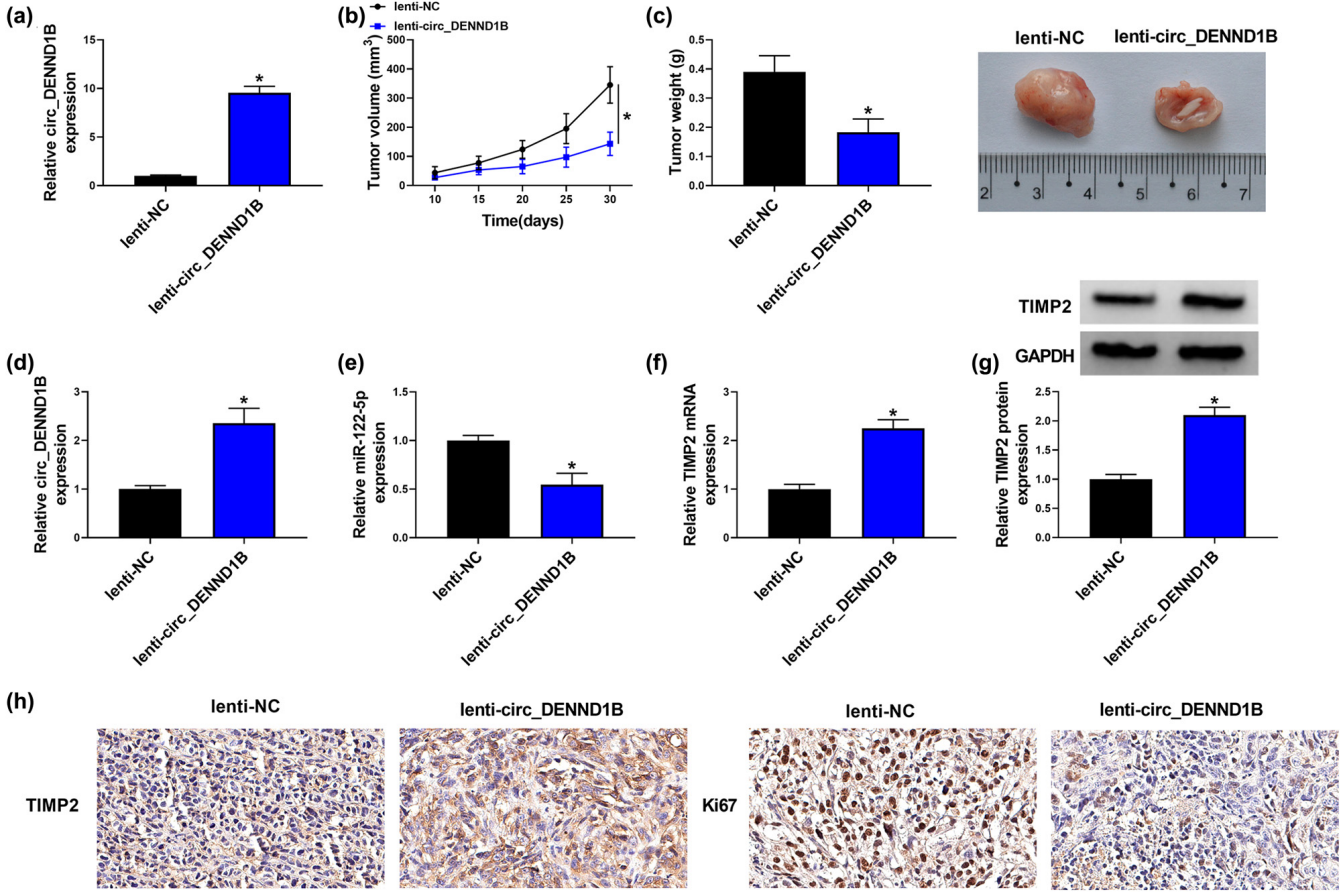
Overexpressed circ_DENND1B inhibits tumor growth *in vivo*. (a) qRT-PCR detected the expression of TIMP2. (b and c) Tumor volume and weight after circ_DENND1B overexpressed *in vivo*. (d–f) qRT-PCR detected the expression of circ_DENND1B, miR-122-5p and TIMP2. (g) Western blot detected the level of TIMP2 protein. (h) IHC detected the positive cells rate of TIMP1 and Ki67. *P < 0.05.

To investigate the effect of circ_DENND1B on tumor *in vivo*, we stably transfected circ_DENND1B into 786-O cells with lentivirus and injected them subcutaneously into mice. As shown in Figure 7a, the overexpressed circ_DENND1B was detected by qRT-PCR. Then, compared with the lenti-NC group, the tumor volume and tumor weight were dramatically reduced in the lenti-circ_DENND1B group (Figure 7b and c). Besides, the expression of circ_DENND1B was increased and the level of miR-122-5p was decreased by circ_DENND1B transfection in mice tumor (Figure 7d and e). And overexpressed circ_DENND1B notably enhanced the level of TIMP2 (Figure 7f and g). Lastly, IHC detection showed that TIMP2- and Ki67-positive cell rates were significantly decreased in the lenti-circ_DENND1B group (Figure 7h). Overall, circ_DENND1B overexpression inhibited the tumor growth of ccRCC *in vivo*.

## 4 Discussion

To prove whether circ_DENND1B played as a ceRNA in ccRCC, we investigated the targeting binding between miR-122-5p and circ_DENND1B/TIMP2 by bioinformatics website prediction and dual-luciferase report analysis. A new circRNA/miRNA/mRNA network regulating ccRCC was discovered. The potential molecular function of circ_DENND1B in ccRCC was revealed by functional experiments, which may provide a new circRNA for the pathogenesis of ccRCC and the exploration of early diagnostic targets in the future.

circRNA has become a hotspot in cancer research and has been found to be abnormally expressed in nasopharyngeal carcinoma [19], ovarian cancer [20], and endometrial cancer [21]. Literature review found that circ_SAR1A [22], circ-EGLN3 [23] and circ_001842 [24] could promote the progression of RCC, while circ_AKT3 [25] and circ_RPL23A [11] exhibited the low expression in RCC and played important roles in cancer suppression. In our study, downregulation of circ_DENND1B in ccRCC was confirmed by the analysis of GSE100186 data and qRT-PCR detection. In addition, our results revealed that upregulation of circ_DENND1B inhibited malignant behavior of ccRCC cells and promoted apoptosis of ccRCC cells. Animal experiments proved that overexpression of circ_DENND1B inhibited the tumor growth of ccRCC.

The effect of circ_DENND1B upregulation on ccRCC cell proliferation was also demonstrated by detecting PCNA protein expression. PCNA exists only in normal proliferating cells and tumor cells, and it has been used as a good indicator for cell proliferation [26]. Tang et al. found that circ_0000515 silencing remarkably impeded the proliferation of cervical cancer cells, and the level of PCNA was significantly reduced [27]. Consistently, circ_DENND1B upregulation reduced the PCNA level. Thus, ccRCC cell proliferation was inhibited.

Similarly, the function of circ_DENND1B on the invasiveness of ccRCC cells was further demonstrated by the detection of EMT-related proteins [28]. EMT is an important indicator of cancer cell migration and invasion, in which E-cadherin is the main protein responsible for cell adhesion junctions and preventing cell metastasis and spread. In contrast, Twist1 promotes tumor cell metastasis [29,30]. Our results showed that overexpression of circ_DENND1 suppressed Twist1 expression and promoted E-cadherin levels, confirming that upregulation of circ_DENND1B repressed the migration and invasive ability of ccRCC cells.

Studies have shown that circRNAs competitively interacted with miRNAs to mediate the biological functions of various cancer cells [31]. Herein, circ_DENND1B was shown to target miR-122-5p. The study of Wang et al. showed that miR-122-5p was upregulated in 786-O cells, and inhibition of miR-122-5p reduced the viability of renal cancer cells and blocked cell cycle progression [15]. Our results were consistent with that miR-122-5p was increased in ccRCC cells. In addition, functional recovery experiments and western blot detection of PCNA, Twist and E-cadherin proteins confirmed that miR-122-5p mimic restored malignant behavior of ccRCC cells inhibited by circ_DENND1B.

It has been reported that miRNA binds to target mRNA 3′UTR and negatively regulates mRNA to regulate cancer progression [8]. TIMP2 was a downstream target of miR-122-5p. Yi et al. presented that EZH2 inhibited the level of tumor suppressor TIMP2 and promoted ovarian cancer cell metastasis through tissue microarray detection [32]. Lu et al. showed that miR-221 targeted TIMP2 to regulate the malignant behaviors of ccRCC cells [17]. Similarly, our results showed that anti-miR-122-5p suppressed the viability and metastasis of ccRCC cells and promoted apoptosis via upregulating the level of TIMP2.

In conclusion, circ_DENND1B suppressed ccRCC cell malignant behaviors by miR-122-5p/TIMP2 axis. These dates demonstrated that circ_DENND1B might be a target for the early diagnosis and treatment of ccRCC.
